# Clinical characteristics and removal approaches of tracheal and bronchial foreign bodies in elders

**DOI:** 10.1038/s41598-024-60307-z

**Published:** 2024-04-25

**Authors:** Ping-Yang Hong, Ling Wang, Yan-Ping Du, Miao Wang, Yi-Yuan Chen, Mao-Hong Huang, Xiao-Bin Zhang

**Affiliations:** 1 Department of Pulmonary and Critical Care Medicine, Zhongshan Hospital of Xiamen University, School of Medicine, Xiamen University, No. 201, Hubin Nan Road, Siming District, Xiamen, Fujian China; 2 The School of Clinical Medicine, Fujian Medical University, Fuzhou, China; 3 Key Clinical Specialty of Fujian Province, Fuzhou, China

**Keywords:** Foreign body, Elders, Flexible bronchoscopy, Tracheobronchial, Risk factors, Respiratory tract diseases

## Abstract

The symptoms of tracheobronchial foreign body in the elderly are not typical, so they are often missed or misdiagnosed. This study aims to depict the clinical characteristics of tracheobronchial foreign body inhalation in the elderly. We retrospectively analysed the clinical data of elder patients (age ≥ 65 years) diagnosed with tracheal and bronchial foreign bodies. The data included age, sex, clinical symptoms, type and location of foreign bodies, prehospital duration, Chest CT, bronchoscopic findings, and frequencies and tools for removing these elderly patients' tracheal and bronchial foreign bodies. All patients were followed up for a half year. Fifty-nine cases were included, of which only 32.2% had a definite aspiration history. Disease duration > 30 days accounted for 27.1% of the patients. 27.1% of the patients had a history of stroke, and 23.8% had Alzheimer's Disease. Regarding clinical symptoms, patients mainly experience cough and expectoration. The most common CT findings were abnormal density shadow (37.3%) and pulmonary infiltration (22.0%). Under bronchoscopy, purulent secretions were observed in 52.5% of patients, and granulation tissue hyperplasia was observed in 45.8%. Food (55.9%) was the most common foreign object, including seafood shells (5.1%), bones (20.3%), dentures (18.6%), and tablets (20.3%). The success rate of foreign body removal under a bronchoscope was 96.7%, 28.8% of the foreign bodies were on the left and 69.5% on the right. 5.1% of the elderly patients required rigid bronchoscopy, and 6.8% required two bronchoscopies. In elderly cohorts, tracheal foreign bodies are obscured by nonspecific clinical presentations and a paucity of aspiration history, challenging timely diagnosis. Predominantly constituted by food particles, with a notable predilection for the left bronchial tree, these cases demand skilled bronchoscopic management, occasionally requiring sophisticated approaches for successful extraction.

## Introduction

Even relatively rare in adults, the incidence of tracheal and bronchial foreign bodies becomes a critical concern in the elderly population. It shows a worrying upward trend with advancing age^1,2^. However, due to their atypical symptoms, these conditions are frequently misdiagnosed as chronic obstructive pulmonary disease and asthma, leading to complications such as obstructive pneumonia and bronchiectasis/stenosis^3^. Tracheal and bronchial foreign bodies are often tricky in diagnosis and differential diagnosis due to significant differences in clinical features, lack of characteristic imaging manifestations, unclear aspiration history, and atypical symptoms. If not timely, treatment can cause severe acute and chronic complications. The bronchoscope is very important in diagnosing and treating the trachea and bronchial foreign body, and its clinical value has been proven in many studies^4,5^. There is scant literature specifically addressing tracheal foreign bodies in the elderly. However, the incidence of tracheal foreign bodies in this population is high, with numerous complications and a significant mortality rate, necessitating clinical attention. In this study, demographic, clinical characteristics, and endoscopic diagnosis and treatment data of 59 elderly patients with trachea and bronchial foreign bodies were retrospectively analysed, aiming to provide more clinical experience for the bronchoscopic diagnosis and treatment.

## Methods

### Research subjects

All methods were carried out following relevant guidelines and regulations. With approval from the Ethics Committee of the Zhongshan Hospital of Xiamen University, Fujian, China, a retrospective review was conducted in the Department of Pulmonary and Critical Care Medicine in the Zhongshan Hospital of Xiamen University. All elder patients (age ≥ 65 years) diagnosed with the trachea and bronchial foreign bodies from December 2017 to June 2022 were enrolled.

### Data collection

We recorded the demographic information, choking history, type of foreign body, radiologic findings, and location of foreign body for 59 patients.

All patients underwent elective or emergent bronchoscopy for aspiration, a foreign body on imaging, and chronic cough. Bronchoscopy was carried out while the patient received either mechanical or spontaneous ventilation. All patients were given 8 ml of 2% lidocaine solution for 15–20 min of atomised inhalation anaesthesia before surgery, combined with intravenous anaesthesia when necessary. Some patients were treated with rigid bronchoscopy. Suction, forceps, or a wire basket that was put via the bronchoscope channel were all used to try and remove foreign objects. Additional procedures, such as rigid bronchoscopy, surgery, or transfer, were carried out in the event of failure. Patients were watched for adverse side effects after the aspirated material was removed.

### Statistical analysis

Categorical variables were expressed as n (%), and continuous variables were expressed as median (IQR, interquartile range). All data analyses were done in SPSS software (version 26.0, IBM, Chicago, USA). All images are drawn through GraphPad Prism (version 8.0.2).

### Ethics approval and consent to participate

The experimental protocol was established, according to the ethical guidelines of the Helsinki Declaration and was approved by the Human Ethics Committee of Zhongshan Hospital of Xiamen University, Xiamen, China. Written informed consent was obtained from individual or guardian participants.

## Results

### General characteristics of elders with tracheal and bronchial foreign bodies

**Figure 1:**
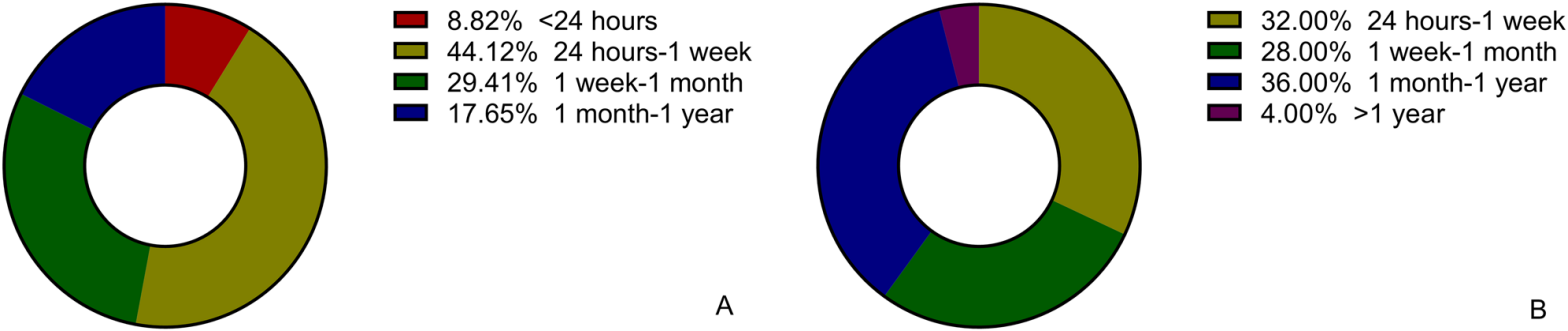
Relationship between place of residence and time elapsed between aspiration or symptoms and admission. Different lengths of time are represented in different colors (n = 59). (**A**) Urban area. (**B**) Rural area.

In total, 59 elderly patients diagnosed with tracheal and bronchial foreign bodies and treated by bronchoscopy were enrolled in our study. The ratio of males (39) to females (20) was 1.95:1, underscoring a male predominance and a higher incidence in the elderly. The age distribution ranged from 65 to 86 years, with a median age of 72 (IQR 67–80) years; elders aged 65 to 75 years accounted for 61.0% (36/59), and those older than 75 years accounted for 39.0% (23/59) of patients. The analysis reveals a broad duration spectrum for foreign body presence, ranging from less than a day to over a year, with a median of one week. A notable fraction of the cohort had a recorded history of aspiration. The examination of comorbidities illustrated a significant prevalence of hypertension alongside other conditions such as stroke and Alzheimer's disease. Symptomatically, cough and sputum were prevalent, with most patients being administered antibiotics, primarily β-lactam types, reflecting a common therapeutic approach. The duration of antibiotic use varied, with a significant portion extending to a week, highlighting the clinical management practices in these cases (Table 1). There were 34 (57.6%) patients from urban areas and the remaining 25 (42.4%) from rural areas (Table 1). Overall, urban patients took less time to see a doctor after developing symptoms than rural patients (Fig. 1).

**Table 1:** The characteristics of patients with tracheal and bronchial foreign bodies.

Characteristics	Numbers (n/%)
Gender (male/female)	39/20
Age	
65 years ≤ age < 75 years	36 (61.0)
Age ≥ 75 years	23 (39.0)
Median age, years	72 (67–80)
Duration	
< 24 h	3 (5.1)
− 1 week	23 (39.0)
− 1 month	17 (28.8)
− 1 year	15 (25.4)
> 1 year	1 (1.7)
Median duration, days	7 (3–30)
History of aspiration	
Positive	19 (32.2)
Negative	40 (67.8)
Comorbidities	
Diabetes	3 (5.1)
Hypertension	25 (42.4)
Stroke	16 (27.1)
Alzheimer's disease	14 (23.8)
Vocal cord paralysis	2 (3.4)
Symptoms	
Cough	40 (67.8)
Sputum	34 (57.6)
Fever	16 (27.1)
Hemoptysis	6 (10.2)
Dyspnea	17 (28.8)
Residence	
Urban area	34 (57.6)
Antibiotics	
Used	52 (88.1)
Not used	7 (11.9)
Types of antibiotics (n = 52)	
β-lactam antibiotics	38 (73.1)
Quinolones	7 (13.5)
Other antibiotics	2 (3.9)
Combination therapy	5 (9.6)
Duration of antibiotic use (n = 52)	
< 3 days	5 (9.6)
− 1 week	27 (51.9)
− 2 week	13 (25.0)
> 2 week	7 (13.5)

Duration = Time elapsed between aspiration/symptoms and admission.

### Chest CT and bronchoscopic findings of tracheal and bronchial foreign bodies

Chest CT scans in patients with tracheal and bronchial foreign bodies revealed diverse findings: 15.3% normal scans, 22% pulmonary infiltration, 11.9% atelectasis, 10.2% radio-opaque bodies, 37.3% abnormal density shadows, and 3.4% space-occupying lesions. Bronchoscopic examinations frequently showed granulation tissue hyperplasia, erosions, and purulent secretions, reflecting the complexity of these cases. These results highlight the varied presentations of airway foreign bodies, emphasizing the need for thorough diagnostic strategies. (Table 2).

**Table 2:** Chest CT and bronchoscopic findings of tracheal and bronchial foreign bodies.

Items	Numbers	Percentages (%)
Chest CT findings		
Normal	9	15.3
Pulmonary infiltration	13	22.0
Atelectasis	7	11.9
Radio-opaque foreign bodies	6	10.2
Abnormal density shadow	22	37.3
Space-occupying lesions	2	3.4
Bronchoscopic findings		
Hyperplasia of granulation tissue	27	45.8
Erosion	21	35.6
Purulent secretion	31	52.5
Scar	8	13.6

### Types and location of tracheal and bronchial foreign bodies

**Figure 2:**
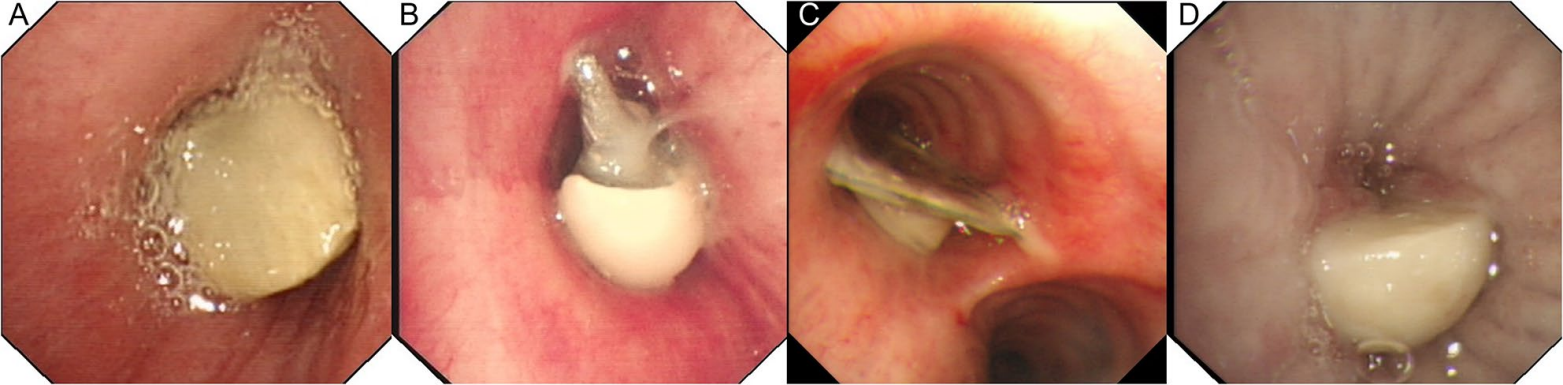
Typical foreign bodies. (**A**) Bones. (**B**) Denture. (**C**) Pill. (**D**) Peanut kernel.

**Figure 3:**
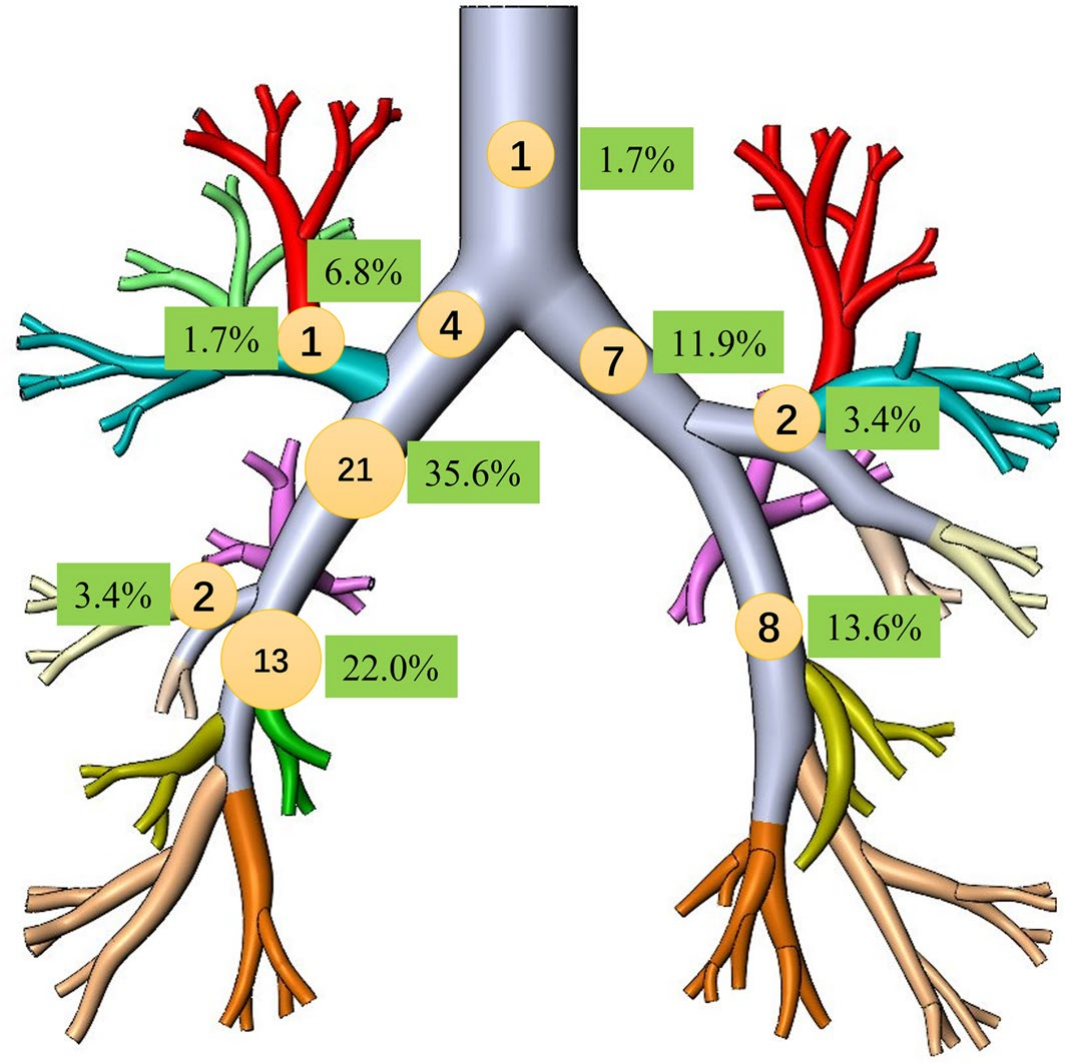
Sites of foreign bodies. Diagrams showing sites of foreign bodies in all cases (n = 59). Numbers in circles are case numbers. Numbers in the green background are percentages.

The study reveals a variety of tracheal and bronchial foreign bodies, notably pills and dentures. Food particles, particularly prune kernels (13.6%) and chicken bones (8.5%), were significant, highlighting dietary risks (Fig. 2). A majority of foreign bodies were located in the right middle bronchus (35.6%) and right lower lobar bronchus (22.0%), suggesting anatomical susceptibilities. Bronchoscopic detection was highest in the right bronchus (69.5%), followed by the left bronchus (28.8%) and trachea (1.7%). These findings suggest trends in airway foreign body incidents, informing clinical preventive and management approaches (Table 3) (Fig. 3).

**Table 3:** Types and location of tracheal and bronchial foreign bodies.

Items	Numbers (%)
Types	
Foods	
Chicken bone	5 (8.5)
Fish bone	3 (5.1)
Shell of crab	2 (3.4)
Bone of pig	2 (3.4)
Prune kernel	8 (13.6)
Meatball	3 (5.1)
Groundnut kernels	5 (8.5)
Lobster shell	1 (1.7)
Unclassifiable bones	4 (6.8)
Denture	11 (18.6)
Pill	12 (20.3)
Metallic foreign body	1 (1.7)
Unknown foreign body	2 (3.4)
Location	
Trachea	1 (1.7)
Left main bronchus	7 (11.9)
Left upper lobar bronchus	2 (3.4)
Left lower lobar bronchus	8 (13.6)
Right main bronchus	4 (6.8)
Right upper lobar bronchus	1 (1.7)
Right middle bronchus	21 (35.6)
Right middle lobar bronchus	2 (3.4)
Right lower lobar bronchus	13 (22.0)

### Removal approaches of tracheal and bronchial foreign bodies

The study primarily utilized general anesthesia for removing tracheal and bronchial foreign bodies in 83.1% of instances, with sedation and local anesthesia less common at 11.9% and 5.1%, respectively. Rigid bronchoscopy was applied in 5.1% of cases, whereas flexible bronchoscopy was favored in 94.9%, reflecting a preference for general anesthesia coupled with flexible bronchoscopy for most extractions. The Allis clamp was the most frequently used extraction tool. The high success rate of single-session bronchoscopic removal underscores the efficacy of these methodologies. (Table 4).

**Table 4:** Removal approaches of tracheal and bronchial foreign bodies.

Items	Numbers	Percentages (%)
Anesthetic mode		
General anesthesia	49	83.1
Sedation and local anesthesia	7	11.9
Local anesthesia	3	5.1
Types of bronchoscope		
Rigid bronchoscope	3	5.1
Flexible bronchoscope	56	94.9
Tools		
Biopsy forceps	13	22.8
Allis clamp	30	52.6
Net basket	3	5.3
Alligator grasping forceps	4	7.0
Grasping forceps under rigid bronchoscope guidance	3	5.3
Combination of two tools	3	5.3
Combination of three tools	1	1.8
Frequency of bronchoscopy		
One	55	93.2
Two	4	6.8

## Discussion

**Figure 4:**
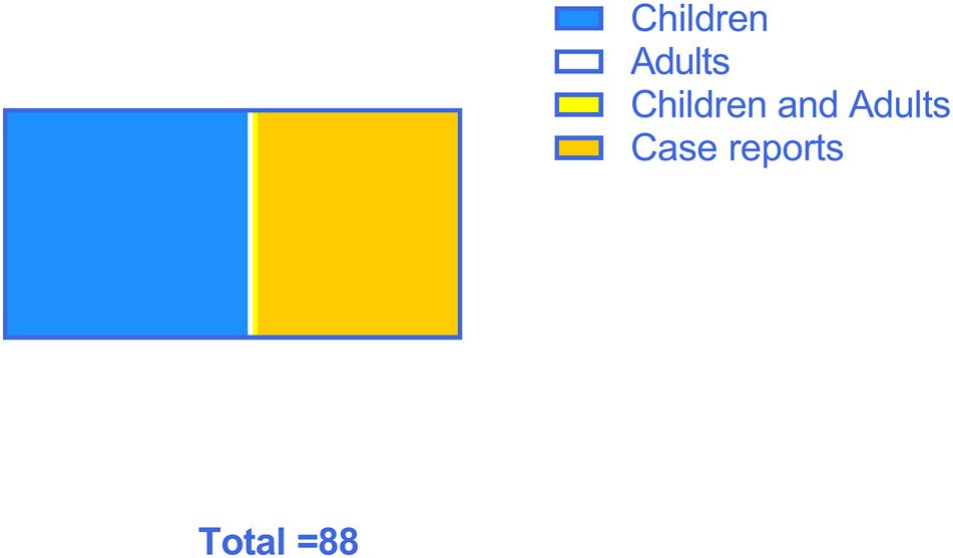
Summary of literature on tracheobronchial foreign bodies in the last five years.

With advancing age, tracheal and bronchial foreign body aspiration becomes increasingly prevalent^6,7^. It is often misdiagnosed or missed due to the lack of clear aspiration history, atypical symptoms, and characteristic imaging manifestations, leading to acute and chronic complications such as asphyxia, obstructive pneumonia, pulmonary atelectasis, and airway stenosis^5^. Following a comprehensive review of the literature on tracheal foreign bodies over the past five years, utilizing databases such as PubMed and Web of Science, we have synthesized the data regarding publications across different age demographics. Notably, case reports constitute nearly half of the publications within this timeframe. Among the remaining research articles, a predominant focus is observed on pediatric cases. The accompanying figure highlights the absence of studies involving older patient populations in the extant literature (Fig. 4 and Supplementary S1). We collected data from our hospital and identified 59 tracheal and bronchial foreign body cases occurring in elderly patients in the past five years. We analysed the data of these cases to understand the clinical characteristics of the trachea foreign body in the elderly.

In most of the older adults in our study, the time from the onset of symptoms to the hospital was between three days and one month. Our study found that older people in urban areas received treatment more promptly than those in rural areas. A study of foreign bodies in children had similar findings. The study found that children in urban areas were seen by doctors faster than those in rural areas^8^.

Our study found that the lower proportion of the elderly with definite aspiration history may be related to the reduced pharyngeal reflex in this population. A study of 234 patients compared the characteristics of tracheobronchial foreign bodies in different age groups and found that the proportion of the elderly with a definite history of aspiration was lower than that of the non-elderly group^8^. We anticipate an increase in predisposing diseases, including stroke, Parkinson's disease, and motor neuron disease, which will increase tracheal and bronchial foreign bodies.

Patients with respiratory problems may also have other medical disorders that may present with symptoms similar to those of tracheal and bronchial foreign bodies, such as conditions that affect neurological state or hinder deglutition^6^. In this study, the most common symptoms of patients with tracheal and bronchial foreign bodies were cough, phlegm, and dyspnea, but these symptoms were not specific. Some patients may have obstructive lesions such as abnormal density shadow, atelectasis, and pulmonary infiltration due to intrinsic foreign bodies, including sputum plug^9^. Therefore, diagnosing tracheal and bronchial foreign bodies depends significantly on history and clinical characteristics^7^. The manifestations under bronchoscopy are mainly purulent secretions^10^ and granulation tissue hyperplasia. Bronchoscopic examination often reveals significant granulation tissue growth, indicating a prolonged duration of the foreign body presence, complicating removal. Our team usually takes comprehensive measures to remove refractory tracheal foreign bodies. High-frequency electrosurgical units can cauterize the granulation tissue around foreign bodies, loosening the foreign object. The cryoprobe can freeze together with the foreign body, forming a single unit, allowing multiple small foreign bodies to be removed at once. Balloons can dilate narrowed airways, facilitating the removal of foreign bodies.

The types of foreign bodies in the trachea and bronchus were mainly food, and bone accounted for a large proportion, similar to previous literature reports^11^. This may be related to the lack of adequate care when the elderly are eating. At the same time, the types of foreign bodies have regional characteristics. People in coastal areas of Xiamen mostly eat seafood, so many seafood foreign bodies account for a relatively high proportion. In this study, dentures were also one of the components of foreign body inhalation. Adult patients older than 65 are more likely to experience foreign body aspiration^12,13^. Pills are also one of the components of inhaling foreign objects in the elderly. According to estimates, 7% of foreign body aspirations involve pills^14,15^. The right bronchus, particularly the mainstem or divisions of the right lower lobe bronchus, is the most often aspirated site in adults^16–18^. Interestingly, our study also found that some elderly foreign bodies were in the left bronchus. This may be related to the fact that these older people are unresponsive, fed by their families, and lie on their left side.

In 96.7% of our patients, bronchoscopy effectively eliminated tracheal and bronchial foreign bodies^4^. Foreign body forceps, biopsy forceps, and foreign body nets are commonly used as auxiliary tools for foreign body removal under bronchoscopy^19^. Most foreign bodies can be removed with a single tool such as an allis clamp, biopsy forceps, or alligator grasping forceps. For complex foreign bodies, it is necessary to combine with other bronchoscopic treatments^5^. Some patients even need rigid bronchoscopy for treatment because of the presence of foreign bodies for too long.

In conclusion, elderly patients with tracheobronchial foreign bodies often present with multiple comorbidities, a protracted disease course, and a notable absence of cough history, with nearly one-third remaining asymptomatic. The prolonged necessity for anti-infection measures further complicates management. Unlike pediatric cases, elderly patients are more likely to have food particles than metal objects as foreign bodies, posing detection challenges via CT imaging. Additionally, while the right trachea is a common site for foreign body lodgment, a significant proportion occurs in the less accessible left trachea. Given the advanced age, the presence of multiple underlying conditions, the atypical clinical and radiological presentations, and the heightened demands on endoscopic techniques and anesthesia, we emphasize the critical importance of vigilance towards tracheobronchial foreign bodies in the elderly, especially as China transitions into an aging society. This perspective underscores the need for tailored diagnostic and therapeutic approaches in this vulnerable population.

### Supplementary Information

Supplementary Information.

## Data Availability

All data generated or analysed during this study are included in this published article.

## References

[1] Takenaka M, Hanagiri T, Ono K (2011). Management of patients with bronchial foreign bodies. J. UOEH..

[2] Boyd M, Watkins F, Singh S (2009). Prevalence of flexible bronchoscopic removal of foreign bodies in the advanced elderly. Age Ageing..

[3] Duan L, Chen X, Wang H, Hu X, Jiang G (2014). Surgical treatment of late-diagnosed bronchial foreign body aspiration: A report of 23 cases. Clin. Respir. J..

[4] Miyashita, Y., Takagi, H., Okamoto, S., *et al.* Treatment of flexible bronchoscopy for bronchial foreign bodies, a single-center investigation. *B37. Interventional Pulmonary: Case Reports I*: American Thoracic Society A3180 (2018).

[5] Yu S-J, Zhu W-J, Zhu H, Yang S, Luo F-M (2021). Retrospective analysis of tracheobronchial foreign bodies in 234 cases of different ages. Int. Respir. J..

[6] Boyd M, Chatterjee A, Chiles C, Chin R (2009). Tracheobronchial foreign body aspiration in adults. S. Med. J..

[7] Ulas AB, Aydin Y, Eroglu A (2022). Foreign body aspirations in children and adults. Am. J. Surg..

[8] Gan W, Xiao N, Feng Y (2021). Clinical analysis of tracheobronchial foreign body aspiration in children: A focus on external and intrinsic factors. BMC Surg..

[9] Tanahashi M (2022). Tracheobronchial foreign body. Kyobu Geka..

[10] da Trindade JGA, Ananias MLB, Fontes CAP (2022). Lung abscess in adult with foreign body (tooth). Am. J. Med..

[11] Jang G, Song JW, Kim HJ, Kim EJ, Jang JG, Cha SI (2022). Foreign-body aspiration into the lower airways in adults; multicenter study. PLoS One..

[12] Soroudi A, Shipp HE, Stepanski BM (2007). Adult foreign body airway obstruction in the prehospital setting. Prehosp. Emerg. Care..

[13] Hou R, Zhou H, Hu K (2016). Thorough documentation of the accidental aspiration and ingestion of foreign objects during dental procedure is necessary: Review and analysis of 617 cases. Head Face Med..

[14] Bowden ET, Smith P, Dwyer KM (2021). Pill aspiration: An under-recognised clinical entity. Med. J. Aust..

[15] Mehta AC, Khemasuwan D (2014). A foreign body of a different kind: Pill aspiration. Ann. Thorac. Med..

[16] Blanco Ramos M, Botana-Rial M, García-Fontán E, Fernández-Villar A, Gallas TM (2016). Update in the extraction of airway foreign bodies in adults. J. Thorac. Dis..

[17] Mi W, Zhang C, Wang H (2015). Measurement and analysis of the tracheobronchial tree in Chinese population using computed tomography. PLoS One..

[18] Liu X, Ni F, Guo T (2022). Risk factors associated with radiolucent foreign body inhalation in adults: A 10-year retrospective cohort study. Respir. Res..

[19] Hewlett JC, Rickman OB, Lentz RJ, Prakash UB, Maldonado F (2017). Foreign body aspiration in adult airways: Therapeutic approach. J. Thorac. Dis..

