# Supplementary material for: Clinical characteristics and removal approaches of tracheal and bronchial foreign bodies in elders
Source: Sci Rep. 2024 Apr 25;14:9493. doi: 10.1038/s41598-024-60307-z (PMC11045842; doi:10.1038/s41598-024-60307-z)
Supplement: Supplementary file 1 — Supplementary Information. [file 41598_2024_60307_MOESM1_ESM.docx]

Supplementary 1 Summary of literature on tracheobronchial foreign bodies in the last five years*

| Types | Titles |
| --- | --- |
| Case reports | Risk factor analysis of bronchospasm after tracheobronchial foreign body removal: Cases report and literature review (STROBE)^1^ |
|  | Tracheobronchial Foreign Body Aspiration Diagnosed with Electrical Impedance Tomography^2^ |
|  | Use of Dormia Basket for Retrieval of Tracheobronchial Foreign body^3^ |
|  | Subcutaneous Emphysema: An Outlandish Hint to an Impacted Tracheobronchial Foreign Body^4^ |
|  | 'PulmoNAILy' injury: surgical management of rare tracheobronchial foreign body aspiration in an adult^5^ |
|  | Extremely Preterm Neonate with a Tracheobronchial Foreign Body: A Case Report^6^ |
|  | Long-standing undiagnosed foreign body aspiration with concomitant pulmonary tuberculosis in an Immunocompetent man^7^ |
|  | A Patient With a Foreign Body in Mediastinum That Penetrated Into the Bronchus^8^ |
|  | Anesthetic Management in a 4-Year-Old Child Undergoing Removal of a Gemstone Tracheobronchial Foreign Body^9^ |
|  | Unusual longstanding intrabronchial foreign body masquerading as intractable bronchial asthma in an adult: Case report and literature review^10^ |
|  | Pediatric Rigid Bronchoscopy for Tracheobronchial Foreign Bodies in Covid Times: Short Personal Experience^11^ |
|  | A Case of Migratory Foreign Body in Left Bronchus -Scarf Pin^12^ |
|  | Neglected Foreign Body Aspiration Mimicking Lung Cancer: A Case Report^13^ |
|  | Distal Airway Aspirated Metallic Foreign Body, Case Report of Spontaneous Expectoration^14^ |
|  | Cyanoacrylate glue foreign body after CT-guided localization of a pulmonary nodule during video-assisted thoracoscopic surgery: a case report^15^ |
|  | Concealed Foreign Body Shrouding Airway Mimicking Mass Causing Extubation Failure, Hypoxia, and Stridor^16^ |
|  | Late Diagnosis of Foreign Body Aspiration in Adults: Case Series and Review of the Literature^17^ |
|  | Anesthetic management in a 1-year-old child undergoing removal of a large metal tracheobronchial foreign body^18^ |
|  | Spontaneous expulsion of an intrabronchial sharp metallic foreign body and migration to the gastrointestinal tract at Muhimbili National Hospital: Case report and literature review^19^ |
|  | Foreign Body Aspiration Mimicking an Endobronchial Neoplasm: A Case Report and Review of the Literature^20^ |
|  | Tracheobronchial tooth and dental prosthesis aspirations: 15 cases^21^ |
|  | Catheter-Guided Basket Removal of a Difficult-to-Reach Pediatric Airway Foreign Body^22^ |
|  | [A neglected bronchial foreign body for 23 years]^23^ |
|  | Diagnosis of a missed bronchial foreign body in an 8-year-old girl: a rare case report^24^ |
|  | Pneumonectomy in a child due to belated diagnosis of foreign body aspiration: a case report^25^ |
|  | Flexible Bronchoscopy as the First-Line Strategy for Extraction of Tracheobronchial Foreign Bodies^26^ |
|  | Apneic anesthesia with THRIVE for pediatric bronchial foreign body removal: A case series^27^ |
|  | Paradoxical cause of weaning failure: Post tracheostomy foreign body causing non-resolving pneumonia with worsening failure to wean^28^ |
|  | Importance of patient history in diagnosis of foreign body aspiration in children: Two case reports^29^ |
|  | A rare complication of pica: Stone aspiration with severe respiratory distress^30^ |
|  | Tension Pneumothorax During Rigid Bronchoscopy for Chronic Foreign Body Removal in a Child: A Case Report^31^ |
|  | Flexible Bronchoscopy Combined with Rigid Bronchoscopy for Treatment of Scarring in the Bronchus Caused by a Foreign Body^32^ |
|  | Scarf pin inhalation; presentation and management; a case series^33^ |
|  | Elevated carcinoembryonic antigen and bronchial obstruction caused by a rotten vegetable leaf mimic lung cancer: A case report^34^ |
|  | Fatal Choking Due to a Slug: A Death Driven by Folklore^35^ |
|  | Tracheobronchopathia osteochondroplastica: a case report highlighting the importance of clinico-radiologic correlation^36^ |
|  | Ex-aspirated: A Case of Dental Product Aspiration With Retrieval Methodology and Current Review^37^ |
|  | Left destroyed lung caused by a pen cap in the left lower lobe bronchus "swallowed" 25 years ago^38^ |
|  | A combination of flexible and rigid bronchoscopy in the successful removal of a residual fish bone from a peripheral bronchus: A case report^39^ |
|  | Surgical Treatment of Lung Abscess Due to an Awn Aspiration in a 9-Year-Old Child: A Case Report^40^ |
| Children | Tracheobronchial foreign body aspiration in children: A retrospective single-center cross-sectional study^41^ |
|  | Oxygen desaturation and time burden during tracheobronchial endoscopy for suspected foreign body in toddlers^42^ |
|  | Pediatric anesthetic for tracheobronchial foreign body extraction: A survey of practice in France^43^ |
|  | Clinical analysis of tracheobronchial foreign body aspiration in children: a focus on external and intrinsic factors^44^ |
|  | Characteristics of correct diagnosis versus misdiagnosis of paediatric tracheobronchial foreign body^45^ |
|  | Tracheobronchial Foreign Bodies in Children: Experience From 1,328 Patients in China^46^ |
|  | Tracheobronchial Foreign Body in Small Children: The Combination of Flexible Bronchoscopy and the Urology Stone Retrieval Basket^47^ |
|  | Application of rigid bronchoscopy for emergent removal of tracheobronchial foreign body in paediatric cases: a learning curve study^48^ |
|  | Challenges in Management of Tracheobronchial Foreign Bodies with Delayed Presentation: An Institutional Experience^49^ |
|  | Risk factors for lower respiratory tract infection in children with tracheobronchial foreign body aspiration^50^ |
|  | [Analyses of diagnosis and treatment of foreign body aspiration in children with tracheobronchial variations]^51^ |
|  | A Tertiary Care Centre Experience on the Management of Paediatric Tracheobronchial Foreign Body During the COVID 19 Pandemic^52^ |
|  | Tracheobronchial Foreign Bodies: The Importance of Timely Intervention and Appropriate Collaboration^53^ |
|  | Management of anesthesia and complications in children with Tracheobronchial Foreign Body Aspiration^54^ |
|  | The Transition of Pediatric Tracheobronchial Foreign Body Cases in the Past 36 Years: A Retrospective Single-Center Study in Japan^55^ |
|  | Different Modalities Used in the Art of Managing Tracheobronchial Foreign Bodies^56^ |
|  | Predictors for intraoperative heart failure in children undergoing foreign-body removal^57^ |
|  | Foreign Body Aspiration in Children and Emergency Rigid Bronchoscopy: A Retrospective Observational Study^58^ |
|  | Characteristics and Treatment of Pediatric Tracheobronchial Foreign Bodies: A Retrospective Analysis of 715 Cases^59^ |
|  | Influential factors for visit time for tracheobronchial foreign bodies in pediatrics^60^ |
|  | Risk Factors for Lower Respiratory Tract Infection Associated With Tracheobronchial Foreign Body Aspiration in Children^61^ |
|  | Can Clinical and Radiological Diagnosis Reduce the Need for Bronchoscopy in Pediatric Tracheobronchial Foreign Body Aspiration Cases?^62^ |
|  | Value of multi-slice spiral computed tomography for diagnosis of tracheobronchial foreign body aspiration in children: 5-year retrospective study^63^ |
|  | Foreign body aspiration in children with negative multi-detector Computed Tomography results: Own experience during 2011-2018^64^ |
|  | Surgical Outcome of Treatment of Tracheobronchial Foreign Bodies in Children: A Retrospective Analysis^65^ |
|  | A single-center experience of pediatric foreign-body aspiration: A retrospective 4-year case series^66^ |
|  | PEANUTS: a national survey on the management of pediatric tracheobronchial foreign bodies^67^ |
|  | Interventional therapy via flexible bronchoscopy in the management of foreign body-related occlusive endobronchial granulation tissue formation in children^68^ |
|  | [Analysis of related factors of secondary pulmonary infection in children with tracheobronchial foreign body]^69^ |
|  | The Utility of Endoscope-Assisted Rigid Bronchoscopy in Pediatric Airway Foreign Body Removals^70^ |
|  | Correlative Factors for Types of Tracheobronchial Foreign Bodies Encountered in Children^71^ |
|  | Comprehensive Analysis of the Diagnosis and Treatment of Tracheobronchial Foreign Bodies in Children^72^ |
|  | Management of Tracheo-Bronchial Foreign Bodies in Children: Our Experience^73^ |
|  | A Novel Two Surgeons Approach to Bronchial Foreign Body Removal by Optical Instruments- An 11 Years Institutional Experience^74^ |
|  | Evaluate the Role of Rigid Bronchoscopy in Tracheo-Bronchial Foreign Bodies^75^ |
|  | Title-Optical Forceps: The Real Boon for Surgeon, Residents and Patients with Foreign Body Aspiration^76^ |
|  | Risk factors for difficult removal of tracheobronchial foreign bodies in children by rigid bronchoscopy^77^ |
|  | Pediatric Airway Foreign Bodies and Their Management by Rigid Bronchoscopy: How I Experienced^78^ |
|  | Endoscopic removal of tracheobronchial foreign bodies: results on a series of 51 pediatric patients^79^ |
|  | [Applicability of a clinical score in patients with suspected foreign body aspiration]^80^ |
|  | Efficacy of premedication with intranasal dexmedetomidine for removal of inhaled foreign bodies in children by flexible fiberoptic bronchoscopy: a randomized, double-blind, placebo-controlled clinical trial^81^ |
|  | Role of Unilateral Vocal Cord Palsy in Causing Recurrent Tracheobronchial Foreign Bodies^82^ |
|  | Frequency of Various Foreign Bodies Retrieved from the Airway During Bronchoscopy in Children: A Pediatric Tertiary Care Center Experience^83^ |
|  | Severe tracheobronchial harm due to lithium button battery aspiration: An in vitro study of the pathomechanism and injury pattern^84^ |
|  | Flexible Bronchoscopic Removal of Foreign Bodies from Airway of Children: Single Center Experience Over 12 Years^85^ |
|  | Cryoextraction via flexible bronchoscopy in children with tracheobronchial obstruction^86^ |
| Adults | Foreign-body aspiration into the lower airways in adults; multicenter study^87^ |
| Children and Adults | Utility of Optical Forceps and Tracheotomy for Tracheobronchal Foreign Body^88^ |

*The data is available as of February 12, 2024

References

1. Cui Y, Shao J, Sun H, Wang X, Zhu Z. Risk factor analysis of bronchospasm after tracheobronchial foreign body removal: Cases report and literature review (STROBE). *Medicine (Baltimore).* 2020;99(52):e23170.

2. Guglielmo RD, Khemani RG. Tracheobronchial Foreign Body Aspiration Diagnosed with Electrical Impedance Tomography. *Case Rep Pediatr.* 2021;2021:9951838.

3. Pradhan P, Parida PK. Use of Dormia Basket for Retrieval of Tracheobronchial Foreign body. *Indian J Otolaryngol Head Neck Surg.* 2023;75(2):1092-1094.

4. Bhatia R, Gupta V, Malik D, Upadhyay K, Reddy NM, Srivastava A. Subcutaneous Emphysema: An Outlandish Hint to an Impacted Tracheobronchial Foreign Body. *Indian J Otolaryngol Head Neck Surg.* 2022;74(Suppl 3):4855-4858.

5. Hon K, Marriott R, Thakur S. 'PulmoNAILy' injury: surgical management of rare tracheobronchial foreign body aspiration in an adult. *ANZ J Surg.* 2021;91(5):E314-e315.

6. Viswanathan S, Rodriguez Prado Y, Chua C, Calhoun DA. Extremely Preterm Neonate with a Tracheobronchial Foreign Body: A Case Report. *Cureus.* 2020;12(4):e7659.

7. Chaurasia S, Prabha BB, Ray S, Chowdhury S. Long-standing undiagnosed foreign body aspiration with concomitant pulmonary tuberculosis in an Immunocompetent man. *J R Coll Physicians Edinb.* 2023;53(2):117-118.

8. Zhai J, Yan X, Ge H, et al. A Patient With a Foreign Body in Mediastinum That Penetrated Into the Bronchus. *Ann Thorac Surg.* 2022;114(4):e237-e239.

9. Shelly, Kumar R, Jayakumar TK, Sinha A, Kumar S. Anesthetic Management in a 4-Year-Old Child Undergoing Removal of a Gemstone Tracheobronchial Foreign Body. *J Indian Assoc Pediatr Surg.* 2023;28(5):448-449.

10. Abraham ZS, Bukanu F, Kimario OM, Kahinga AA. Unusual longstanding intrabronchial foreign body masquerading as intractable bronchial asthma in an adult: Case report and literature review. *Int J Surg Case Rep.* 2021;86:106340.

11. Patigaroo SA, Qazi SM, Ahmad R, et al. Pediatric Rigid Bronchoscopy for Tracheobronchial Foreign Bodies in Covid Times: Short Personal Experience. *Indian J Otolaryngol Head Neck Surg.* 2022;74(Suppl 2):2729-2737.

12. Rajmohan D, P A. A Case of Migratory Foreign Body in Left Bronchus -Scarf Pin. *Indian J Otolaryngol Head Neck Surg.* 2023;75(2):1169-1172.

13. Qureshi AS, Mohamed SA, Mohamed A. Neglected Foreign Body Aspiration Mimicking Lung Cancer: A Case Report. *Cureus.* 2021;13(4):e14566.

14. Al Maary J, Alahmari AS. Distal Airway Aspirated Metallic Foreign Body, Case Report of Spontaneous Expectoration. *Am J Case Rep.* 2020;21:e917608.

15. Deng J, Zeng Z, Liao Y, Zhong H, Zhang H. Cyanoacrylate glue foreign body after CT-guided localization of a pulmonary nodule during video-assisted thoracoscopic surgery: a case report. *BMC Pulm Med.* 2023;23(1):24.

16. Hamid K, Perinkulam Sathyanarayanan S, Devasahayam J. Concealed Foreign Body Shrouding Airway Mimicking Mass Causing Extubation Failure, Hypoxia, and Stridor. *Cureus.* 2022;14(6):e26338.

17. Kara K, Ozdemir C, Tural Onur S, Satici C, Tokgoz Akyil F, Nedime Sokucu S. Late Diagnosis of Foreign Body Aspiration in Adults: Case Series and Review of the Literature. *Respir Care.* 2023.

18. Ren Y, Zhang J, Xin Z. Anesthetic management in a 1-year-old child undergoing removal of a large metal tracheobronchial foreign body. *Pediatr Investig.* 2019;3(3):185-187.

19. Abraham ZS, Kahinga AA, Mapondella KB, Massawe ER, Ntunaguzi D. Spontaneous expulsion of an intrabronchial sharp metallic foreign body and migration to the gastrointestinal tract at Muhimbili National Hospital: Case report and literature review. *Int J Surg Case Rep.* 2020;72:423-425.

20. Purohit K, Grandfield S, Dhamija A, Abbasi A. Foreign Body Aspiration Mimicking an Endobronchial Neoplasm: A Case Report and Review of the Literature. *Cureus.* 2023;15(3):e36105.

21. Eliçora A, Sezer HF, Topçu S, Çardaközü T. Tracheobronchial tooth and dental prosthesis aspirations: 15 cases. *J Cardiothorac Surg.* 2023;18(1):78.

22. Gabinet-Equihua A, Cushing SL, Propst EJ, Gai N, Wolter NE. Catheter-Guided Basket Removal of a Difficult-to-Reach Pediatric Airway Foreign Body. *Laryngoscope.* 2021;131(12):2795-2797.

23. Marouf R, Hamraoui S, Alloubi I. [A neglected bronchial foreign body for 23 years]. *Rev Mal Respir.* 2019;36(8):1002-1007.

24. Ashkan Moslehi M, Mohamadian A. Diagnosis of a missed bronchial foreign body in an 8-year-old girl: a rare case report. *Qatar Med J.* 2021;2021(1):6.

25. Razafimanjato NNM, Ralaivao RA, Ravelomihary TDN, Hunald FA, Rakotovao JLH. Pneumonectomy in a child due to belated diagnosis of foreign body aspiration: a case report. *J Med Case Rep.* 2021;15(1):533.

26. Nunes Caldeira J, Fernandes L, Rodrigues Sousa S, Martins Y, Barata F. Flexible Bronchoscopy as the First-Line Strategy for Extraction of Tracheobronchial Foreign Bodies. *Open Respir Arch.* 2022;4(3):100174.

27. Harde MJ, Kamble T, Ramchandani P. Apneic anesthesia with THRIVE for pediatric bronchial foreign body removal: A case series. *Saudi J Anaesth.* 2023;17(2):239-241.

28. Mangla L, Thote S, Talwar D, Talwar D. Paradoxical cause of weaning failure: Post tracheostomy foreign body causing non-resolving pneumonia with worsening failure to wean. *Respirol Case Rep.* 2022;10(7):e0977.

29. Cui Y, Cui X, Yu T, Zhu Z, Wang X. Importance of patient history in diagnosis of foreign body aspiration in children: Two case reports. *Medicine (Baltimore).* 2019;98(17):e15326.

30. Ekinci F, Yildizdas D, Horoz OO, Kilic S, Gokay N. A rare complication of pica: Stone aspiration with severe respiratory distress. *Niger J Clin Pract.* 2021;24(2):295-298.

31. Kumar A, Shiwalkar N, Aslam H, Persaud P. Tension Pneumothorax During Rigid Bronchoscopy for Chronic Foreign Body Removal in a Child: A Case Report. *Cureus.* 2019;11(9):e5628.

32. Wu L, Sheng Y, Xu X, et al. Flexible Bronchoscopy Combined with Rigid Bronchoscopy for Treatment of Scarring in the Bronchus Caused by a Foreign Body. *Case Rep Med.* 2019;2019:4616298.

33. Abduljabbar MA, Jabir SN, Ahmed OF, et al. Scarf pin inhalation; presentation and management; a case series. *Ann Med Surg (Lond).* 2021;62:73-75.

34. Sun Y, Ge YL, Li LQ, et al. Elevated carcinoembryonic antigen and bronchial obstruction caused by a rotten vegetable leaf mimic lung cancer: A case report. *J Clin Lab Anal.* 2021;35(1):e23579.

35. Atreya A, Ghimire S, Kanchan T, Kunwar S, Shah D. Fatal Choking Due to a Slug: A Death Driven by Folklore. *Wilderness Environ Med.* 2023;34(1):100-102.

36. Rodriguez NJV, Manto JT, Sydiongco-Inocencio PMM, Dela Cruz JMG, Ilustre GMS, Dela Cruz APC. Tracheobronchopathia osteochondroplastica: a case report highlighting the importance of clinico-radiologic correlation. *BJR Case Rep.* 2023;9(6):20230062.

37. Nassif ND, Ubhi M, Kapoor A. Ex-aspirated: A Case of Dental Product Aspiration With Retrieval Methodology and Current Review. *Cureus.* 2023;15(5):e39074.

38. Cheng J, Li C, Corsini EM, Antonoff MB, Hekmat K, Liu L. Left destroyed lung caused by a pen cap in the left lower lobe bronchus "swallowed" 25 years ago. *Ann Transl Med.* 2019;7(22):711.

39. Shao H, Li S, He J, Wu L, Chen Z. A combination of flexible and rigid bronchoscopy in the successful removal of a residual fish bone from a peripheral bronchus: A case report. *Front Pediatr.* 2023;11:1114043.

40. Vlahova A, Antonova Z, Rangelov E, et al. Surgical Treatment of Lung Abscess Due to an Awn Aspiration in a 9-Year-Old Child: A Case Report. *Children (Basel).* 2023;10(6).

41. Ding G, Wu B, Vinturache A, Cai C, Lu M, Gu H. Tracheobronchial foreign body aspiration in children: A retrospective single-center cross-sectional study. *Medicine (Baltimore).* 2020;99(22):e20480.

42. Aubanel S, Izaute G, Gariel C, et al. Oxygen desaturation and time burden during tracheobronchial endoscopy for suspected foreign body in toddlers. *J Clin Monit Comput.* 2021;35(5):1077-1084.

43. Loreau C, Caruselli M, Roncin C, et al. Pediatric anesthetic for tracheobronchial foreign body extraction: A survey of practice in France. *Paediatr Anaesth.* 2023;33(9):736-745.

44. Gan W, Xiao N, Feng Y, et al. Clinical analysis of tracheobronchial foreign body aspiration in children: a focus on external and intrinsic factors. *BMC Surg.* 2021;21(1):108.

45. Wang L, Zhang L, Li C, et al. Characteristics of correct diagnosis versus misdiagnosis of paediatric tracheobronchial foreign body. *Paediatr Child Health.* 2021;26(1):e6-e10.

46. Ding L, Su S, Chen C, Yao H, Xiao L. Tracheobronchial Foreign Bodies in Children: Experience From 1,328 Patients in China. *Front Pediatr.* 2022;10:873182.

47. Ciriaco P, Negri G. Tracheobronchial Foreign Body in Small Children: The Combination of Flexible Bronchoscopy and the Urology Stone Retrieval Basket. *Arch Bronconeumol (Engl Ed).* 2019;55(3):174-175.

48. Bin X, Liu L, Fang Q, Tan SH, Tang AZ. Application of rigid bronchoscopy for emergent removal of tracheobronchial foreign body in paediatric cases: a learning curve study. *J Laryngol Otol.* 2022;136(11):1130-1134.

49. Kaushal D, Goyal A, Soni K, et al. Challenges in Management of Tracheobronchial Foreign Bodies with Delayed Presentation: An Institutional Experience. *Int Arch Otorhinolaryngol.* 2022;26(1):e005-e009.

50. Zhong B, Sun SL, Du JT, et al. Risk factors for lower respiratory tract infection in children with tracheobronchial foreign body aspiration. *Medicine (Baltimore).* 2019;98(10):e14655.

51. Wang WW, Cheng HJ, Li M, et al. [Analyses of diagnosis and treatment of foreign body aspiration in children with tracheobronchial variations]. *Zhonghua Er Bi Yan Hou Tou Jing Wai Ke Za Zhi.* 2019;54(10):760-763.

52. Hameed S, Reddy YM. A Tertiary Care Centre Experience on the Management of Paediatric Tracheobronchial Foreign Body During the COVID 19 Pandemic. *Indian J Otolaryngol Head Neck Surg.* 2022;74(Suppl 2):3473-3480.

53. Chouhan M, Sharma S. Tracheobronchial Foreign Bodies: The Importance of Timely Intervention and Appropriate Collaboration. *Indian J Otolaryngol Head Neck Surg.* 2019;71(Suppl 1):972-975.

54. Karaaslan E, Yildiz T. Management of anesthesia and complications in children with Tracheobronchial Foreign Body Aspiration. *Pak J Med Sci.* 2019;35(6):1592-1597.

55. Takahashi H, Suzuki J, Ikeda R, et al. The Transition of Pediatric Tracheobronchial Foreign Body Cases in the Past 36 Years: A Retrospective Single-Center Study in Japan. *Tohoku J Exp Med.* 2023;261(2):129-137.

56. Hemead HM, Ramadan A, Gaafar AH, Nossier A, Abdelaziz A. Different Modalities Used in the Art of Managing Tracheobronchial Foreign Bodies. *Open Respir Med J.* 2022;16:e187430642206100.

57. Yao X, Zhang L, Zhao G, et al. Predictors for intraoperative heart failure in children undergoing foreign-body removal. *Medicine (Baltimore).* 2021;100(28):e26626.

58. Ghosh D, Mitra R, Mondal S, Kumar S, Sengupta A. Foreign Body Aspiration in Children and Emergency Rigid Bronchoscopy: A Retrospective Observational Study. *Indian J Otolaryngol Head Neck Surg.* 2023;75(3):1533-1541.

59. Yan S, Jiang P, Chen G, et al. Characteristics and Treatment of Pediatric Tracheobronchial Foreign Bodies: A Retrospective Analysis of 715 Cases. *Med Sci Monit.* 2022;28:e937928.

60. Zhou J, Shang WY, Huang ZH, et al. Influential factors for visit time for tracheobronchial foreign bodies in pediatrics. *Eur Arch Otorhinolaryngol.* 2020;277(2):505-509.

61. Lin FZ, Cao W, Xu B, Liu J, Bi J, Fu Y. Risk Factors for Lower Respiratory Tract Infection Associated With Tracheobronchial Foreign Body Aspiration in Children. *Ann Otol Rhinol Laryngol.* 2023;132(10):1228-1232.

62. Al Masaoudi L, Kolethekkat AA, Jose S, Al Abri R. Can Clinical and Radiological Diagnosis Reduce the Need for Bronchoscopy in Pediatric Tracheobronchial Foreign Body Aspiration Cases? *Oman Med J.* 2022;37(4):e409.

63. Shen J, Huang L, Hao C. Value of multi-slice spiral computed tomography for diagnosis of tracheobronchial foreign body aspiration in children: 5-year retrospective study. *Pediatr Int.* 2020;62(10):1184-1188.

64. Qiu W, Wu L, Chen Z. Foreign body aspiration in children with negative multi-detector Computed Tomography results: Own experience during 2011-2018. *Int J Pediatr Otorhinolaryngol.* 2019;124:90-93.

65. Onakpoya UU, Eyekpegha OJ, Ojo OO, Oguns AE. Surgical Outcome of Treatment of Tracheobronchial Foreign Bodies in Children: A Retrospective Analysis. *West Afr J Med.* 2021;38(7):684-688.

66. Dorterler ME, Kocaman OH, Gunendi T, Boleken ME. A single-center experience of pediatric foreign-body aspiration: A retrospective 4-year case series. *Lung India.* 2019;36(3):202-206.

67. Chebib E, Benoit C, Van Den Abbeele T, Teissier N. PEANUTS: a national survey on the management of pediatric tracheobronchial foreign bodies. *Eur J Pediatr.* 2023;182(2):591-600.

68. Li S, Wu L, Zhou J, et al. Interventional therapy via flexible bronchoscopy in the management of foreign body-related occlusive endobronchial granulation tissue formation in children. *Pediatr Pulmonol.* 2021;56(1):282-290.

69. Wen X, Shi J, Cui L, et al. [Analysis of related factors of secondary pulmonary infection in children with tracheobronchial foreign body]. *Lin Chuang Er Bi Yan Hou Tou Jing Wai Ke Za Zhi.* 2019;33(12):1200-1202.

70. Ozdemir S, Surmelioglu O, Tarkan O, Tuncer U, Kiroglu M, Dagkiran M. The Utility of Endoscope-Assisted Rigid Bronchoscopy in Pediatric Airway Foreign Body Removals. *J Craniofac Surg.* 2020;31(2):e217-e219.

71. Su S, Zhang H, Xiao L, Yao H, Ding L. Correlative Factors for Types of Tracheobronchial Foreign Bodies Encountered in Children. *Ear Nose Throat J.* 2023:1455613231167244.

72. Wang Y, Sun Y, Zhang H, Yang X, Song X. Comprehensive Analysis of the Diagnosis and Treatment of Tracheobronchial Foreign Bodies in Children. *Ear Nose Throat J.* 2023;102(10):661-666.

73. Gayen GC, Das D, Ray R, Maitra M. Management of Tracheo-Bronchial Foreign Bodies in Children: Our Experience. *Indian J Otolaryngol Head Neck Surg.* 2022;74(4):536-539.

74. Sikdar A, Mahajan A, Nivsarkar S, Phatak S, Agarwal R. A Novel Two Surgeons Approach to Bronchial Foreign Body Removal by Optical Instruments- An 11 Years Institutional Experience. *Indian J Otolaryngol Head Neck Surg.* 2022;74(4):469-475.

75. Wadhera R, Sehrawat U, Hooda S, Wadhera S. Evaluate the Role of Rigid Bronchoscopy in Tracheo-Bronchial Foreign Bodies. *Indian J Otolaryngol Head Neck Surg.* 2022;74(Suppl 3):5177-5181.

76. Dubey NK, Upadhyay A, Raghuwanshi N, Godha S, Mundra R. Title-Optical Forceps: The Real Boon for Surgeon, Residents and Patients with Foreign Body Aspiration. *Indian J Otolaryngol Head Neck Surg.* 2022;74(Suppl 3):5354-5360.

77. Ding L, Su X, Yang D, Yao H, Xiao L. Risk factors for difficult removal of tracheobronchial foreign bodies in children by rigid bronchoscopy. *Int J Pediatr Otorhinolaryngol.* 2023;168:111539.

78. Patigaroo SA, Mehfooz N, Shafi OM, Qazi SM, Ahmad R. Pediatric Airway Foreign Bodies and Their Management by Rigid Bronchoscopy: How I Experienced. *Indian J Otolaryngol Head Neck Surg.* 2022;74(Suppl 3):6422-6437.

79. De Palma A, Brascia D, Fiorella A, et al. Endoscopic removal of tracheobronchial foreign bodies: results on a series of 51 pediatric patients. *Pediatr Surg Int.* 2020;36(8):941-951.

80. Miró I, Ibáñez V, Carazo E, Couselo M, Vila JJ. [Applicability of a clinical score in patients with suspected foreign body aspiration]. *Cir Pediatr.* 2019;32(4):172-176.

81. Bi Y, Ma Y, Ni J, Wu L. Efficacy of premedication with intranasal dexmedetomidine for removal of inhaled foreign bodies in children by flexible fiberoptic bronchoscopy: a randomized, double-blind, placebo-controlled clinical trial. *BMC Anesthesiol.* 2019;19(1):219.

82. Pincet L, Lambercy K, Sandu K. Role of Unilateral Vocal Cord Palsy in Causing Recurrent Tracheobronchial Foreign Bodies. *Front Pediatr.* 2019;7:282.

83. Chand R, Shaikh M, Khan Y, Qureshi MA, Maheshwari H, Yasir M. Frequency of Various Foreign Bodies Retrieved from the Airway During Bronchoscopy in Children: A Pediatric Tertiary Care Center Experience. *Cureus.* 2020;12(7):e9348.

84. Voelker J, Voelker C, Engert J, Schendzielorz P, Hagen R, Rak K. Severe tracheobronchial harm due to lithium button battery aspiration: An in vitro study of the pathomechanism and injury pattern. *Int J Pediatr Otorhinolaryngol.* 2020;139:110431.

85. Kapoor R, Chandra T, Mendpara H, Gupta R, Garg S. Flexible Bronchoscopic Removal of Foreign Bodies from Airway of Children: Single Center Experience Over 12 Years. *Indian Pediatr.* 2019;56(7):560-562.

86. Truitt BA, Kasi AS, Kamat PP, Fundora MP, Simon DM, Guglani L. Cryoextraction via flexible bronchoscopy in children with tracheobronchial obstruction. *Pediatr Pulmonol.* 2023;58(9):2527-2534.

87. Jang G, Song JW, Kim HJ, Kim EJ, Jang JG, Cha SI. Foreign-body aspiration into the lower airways in adults; multicenter study. *PLoS One.* 2022;17(7):e0269493.

88. Kirti YK, Yashveer JK, Soni S, Ruchi. Utility of Optical Forceps and Tracheotomy for Tracheobronchal Foreign Body. *Indian J Otolaryngol Head Neck Surg.* 2022;74(Suppl 3):5182-5187.
